# Long-acting family planning switching and associated factors among revisit women in Toke Kutaye district of West Shoa Zone, Oromia Region public health facilities, Ethiopia: a mixed methods study

**DOI:** 10.1186/s12905-023-02664-x

**Published:** 2023-09-25

**Authors:** Fayera Teshoma, Eden Girmaye Tefera, Teka Girma, Misganu Teshoma Ragasa, Ephrem Yohannes, Gizachew Abdissa Bulto, Negash Wakgari

**Affiliations:** 1 Guder Hospital, West Shoa Zone, Oromia Region, Guder, Ethiopia; 2 Department of Midwifery, College of Medicine and Health Science, Ambo University, Ambo, Ethiopia; 3 Department of Public Health, College of Medicine and Health Science, Ambo University, Ambo, Ethiopia; 4 Department of Midwifery, Institute of Health Science, Wollega University, Nekemte, Ethiopia

**Keywords:** Contraception, Long-acting family planning, Mixed method, Revisit, Switching, Ethiopia

## Abstract

**Background:**

Switching from a long-acting family planning (LAFP) method to another could lead to an unintended pregnancy. However, the proportions of LAFP method switching and predictable factors are not well addressed. Therefore, the aim of this study was to determine the magnitude of LAFP method switching and associated factors among revisit women. The study also explored the reasons for the LAFP method switching among the revisited women.

**Method:**

A mixed methods study was conducted among 377 reproductive age women attending public health facilities in Toke Kutaye district, West Shoa, Zone, Ethiopia, from 20 May 2021 to 28 July 2021. A systematic random sampling for quantitative and purposive sampling technique for qualitative study was used to select the study participants. A pretested structured questionnaire and in-depth interview were used to determine and explore long-acting family planning switching among revisit women. Data were analysed by Statistical Package for the Social Sciences (SPSS) version 21. Binary logistic regression was conducted to identify the dependent and independent variables at *p*-value < 0.05 along with 95% Confidence Interval (CI) and Adjusted Odds Ratio (AOR). The qualitative data were analysed using thematic analysis.

**Results:**

The magnitude of long-acting family planning method switching was 53.3%. Switching from an implant to other short-acting method was 39.8%, and switching from an intrauterine contraceptive device (IUCD) to other short-acting method was 13.5%. A formal education (AOR, 10.38, 95% CI: 3.48, 30.95), birth spacing (AOR, 5.52, 95% CI: 1.31, 23.33) and perceived infertility (AOR, 11.16, 95% CI: 5.55, 22.45) were factors associated with LAFP switching. The qualitative findings revealed that fear of side effects, lack of adequate information, religion, and misconceptions hinder users from maintaining the LAFP.

**Conclusions:**

The study finds that the proportion of women switching from long-acting family planning was relatively higher than in other studies. The main reasons for LAFP switching were fear of side effects, lack of adequate information specific to LAFP and misconceptions. Therefore, the provision of quality contraceptive counselling by the service providers may mitigate the concern of IUD and implant switching. Furthermore, future prospective research at a larger sample size is needed.

## Introduction

Ensuring effective use of the long-acting reversible contraceptive (LARC) method is imperative for the effectiveness of fertility control. Increasing access to family planning methods is a rewarding strategy for tackling maternal morbidity and mortality [1]. However, the majority of women in low- and middle-income countries die from preventable causes of unsafe abortions and complications related to unwanted pregnancy [2]. Studies have revealed that the multitude of unintended pregnancies are the result of the use of less effective methods, incorrect use, avoidance of use, or inadequately swift switching to an alternative method [3].

Method switching refers to the client changing from one contraceptive method to another method [4]. Long-acting reversible contraceptive method switching has been escalated among users for various reasons, including unfavorable bleeding, dysmenorrhea and planning for a pregnancy [5]. Switching from a contraceptive method to a less effective method increases the risk of unintended pregnancies. Studies have shown that the lack of information about the possible adverse effects and information about other available family planning methods increases the rate of contraceptive discontinuation [6].

The Ethiopian demographic health survey (EDHS) states that the use of modern contraceptives among currently married women has increased from 14% in 2005 to 41% in 2019; from this, injectables (27%) were widely used, followed by implants (9%), IUDs (2%), and pills (2%) [7]. The pooled practice of the long-acting contraceptive method in Ethiopia was 16.6% [8]. The possible reasons for the low usage of long-acting family planning were due to women’s insufficient knowledge, lack of electronic media and inaccessibility of methods at lower levels of health service delivery [9].

The effectiveness of long-acting reversible contraceptive methods is inevitable. However, the LARC method discontinuations and switching escalates in sub-Saharan African countries. In the same manner, the success of family planning services is not only maintained by the number of users but also asserted by correct, consistent, and effective contraceptive use [10]. Correspondingly, a systematic review suggests that if family planning counselling is properly harnessed, it can help to leverage early LARC method outages [11].

Changes in early contraceptive methods within six months of initiation not only decreased client satisfaction but also presented care providers with challenges [4]. Despite the fact that the rate of contraceptive prevalence is increasing rapidly in Ethiopia, long-acting contraceptive method switching remains a challenge. The main reason for method switching was side effects such as heavy menstrual bleeding, weight loss and arm numbness [10]. Most of women switched from long-acting reversible method to a short-acting hormonal method and non-hormonal method [4]. Despite the fact that abrupt removal of long-acting family planning might lead to unintended pregnancies, some studies shows that assisting women in choosing methods enhance continuous method use [12, 13]. Thus, identifying factors that contribute to contraceptive switching can reduce the switching rate, unintended pregnancies, induced abortions, and fertility rates [14].

Most of the studies in Ethiopia evaluated the number of contraceptive users. Nevertheless, the rationale for contraceptive method switching is not widely reported in Ethiopian studies. Therefore, the aim of this study was to determine the magnitude and rationale of long-acting reversible family planning switching among revisit clients.

## Methods

### Study area and period

The study was conducted at public health facilities that provide long-acting family planning services in the Toke Kutaye district of West Shoa Zone, Oromia Region, Ethiopia. Guder is the capital town of the district, which is located 126 km to the west of Addis Ababa, the capital city of Ethiopia. The district has 27 kebeles (the lowest administrative unit in Ethiopia), one hospital, four health centers and 24 health posts. Based on the current data, the population of the district is projected to be approximately 128,259, of which 63,873 are males and 64,386 are females, and the reproductive age of women from 15 to 49 is 23,895 [15]. The study was conducted from 20 May 2021 to 28 July 2021.

### Study design

A mixed research design was employed.

### Study population

Reproductive-age women (15–49 years) who had used the long-acting family planning method at least once, currently used who revisited the study settings during the data collection period were enrolled for quantitative data, and family planning methods providers (Nurses and Midwives) who work in the study health facilities were included for the qualitative study.

### Eligibility criteria

#### Inclusion criteria

Long-acting family planning method users current or previous, age between 15 and 49 years who revisited the selected health facilities during the data collection period and providers were included in the study.

#### Exclusion criteria

Women who did not use any long-acting family planning methods at least once and age below 15 and above 49 years were excluded from this study.

### Sample size determination

#### For quantitative study

The sample size was calculated using the single population proportion formula [(*n* = (Zά/2)^2^p (1—p)/d2)]. The following assumptions were made to calculate the required sample size, i.e., *P* = 40.4% [10], level of significance (α = 0.05), Z α/2 = 1.96 with 95% CI, 5% margin of error (d) tolerated for the *p* value *n* = sample size $n=((Z \alpha )/2)2*P(1-p)$/d^2^.

*n* = (1.96)^2^*0.404(1–0.404)/0.05^2^ *n* = 370, considering a 5% non-respondent rate, the final sample size was = 389.

#### Sampling technique and procedure

The required sample size (*n* = 389) was selected from all five public health facilities in the district which includes Guder hospital, Guder health center, Goro Sole health center, Maruf health center and Toke health center. Two months prior to data collection, a total of 1,024 revisit long-acting family planning clients had attended those health facilities. Of these, 32 clients were at Guder hospital, 370 were at Guder health center, 300 at Goro Sole health center, 260 were at Maruf health center and 62 were at Toke health center. Accordingly, by using proportional to size allocation, twelve clients from Guder hospital, 140 clients from Guder health center, 114 clients from Goro Sole health center, 99 clients from Maruf health center and 24 clients from Toke health center were included in this study. Health facilities family planning client registration consisting list of repeat users of LAFP were used as a sampling frame. Then, systematic random sampling method was used to select participants every 3rd (1024/389) interval from those repeat clients who came after using LAFP methods.

#### For qualitative study

A purposive sampling method was used to select the study participants. An in-depth interview was conducted among twelve family planning providers. They were recruited purposively from the selected health facilities.

### Study variables

#### Dependent variable

Long-acting family planning method switching.

#### Independent variables

Socio-demographic factors: age, ethnicity, religion, educational status, marital status, educational status of women and their spouse, occupation, place of residence. Reproductive history: parity, number of children, future fertility plan, and age at first birth. Perceived needs: family planning demands (limiting or spacing), obtain information about family planning methods and user’s family planning perception.

### Operational definition

Long-acting family planning switching: the pattern of switching the contraceptive method from one long-acting (IUCD and implant) to another short-acting method [16].

### Data collection tool and procedures

Data were collected from 20 May 2021 to 28 July 2021 using a semi-structured questionnaire and in-depth interviews. The questionnaire was first prepared in English and then translated to the local language (Afaan Oromo). The second version of the tool was retranslated to the original version by a language expert to evaluate its consistency. The data were collected by recruiting five midwives. The principal investigator supervised the data collection procedures at all study settings. Training was given for the data collectors for two days about the aim of the study, data collection techniques and procedures. For the qualitative study, the key informants (family planning providers) were interviewed in a quiet and confidential room. The in-depth interviews were audio recorded and notes were taken during the field. The interview continued until data saturation was achieved. The audio-recorded interview and field notes were transcribed verbatim immediately after the interview was completed. All the data were placed in a locked cabinet and secured.

### Data quality control

To assure the quality of the data, the questionnaires were pretested on 5% of the sample size other than the study area among women who switched LAFP methods in Ambo General Hospital. Training was given to the data collectors to ensure the accuracy and consistency of the data. The principal investigator spot-checked and reviewed all the completed questionnaires to ensure the completeness and consistency of the collected data. Data entry was performed by the principal investigator to maintain the accuracy of the data. The trustworthiness of the qualitative data, such as the credibility, transferability, dependability and reliability of the data, was rigorously ensured [17–19].

### Data management and analysis

All the questionnaires were checked, coded and entered into Epinfo version 7 and exported to SPSS version 21 software for data analysis. Binary logistic regression analysis was conducted to examine the statistical association between dependent and independent variables. Both bivariate and multivariable logistic regressions were used to identify the associated factors. Variables that had a statistical association in the bivariate logistic regression at *p*-value < 0.25 at 95% CI were entered into a multivariable logistic regression to control the confounding variables [20]. Hence, an adjusted odds ratio (AOR) with a 95% CI at a *p* value < 0.05 was considered to indicate the statistical significance. Finally, the results were presented in the form of tables, figures and text using frequencies and summary statistics such as the mean, standard deviation and percentage to describe the study population in relation to relevant variables.

The audio-recorded interview and field notes were transcribed verbatim immediately after the interview was completed. The audio-recorded interviews and field notes were transcribed verbatim carefully by a local language (Afan Oromo) word by word and arranged with the written notes taken at the time of discussion and interview. Then, the transcribed verbatim was translated to English. The researcher read the whole transcription several times. Then, the transcribed verbatim were imported and organized using NVivo version 12. Then, the data were coded, sorted and analysed thematically. The grouping codes that emerged from the data analysis were presented in themes and then triangulated with the quantitative findings to gain insight into a problem. Finally, the researcher shows how the qualitative data explain the quantitative results.

## Results

### Phase I: Quantitative results

#### Socio-demographic characteristics of the respondents

From 389 samples, those who responded to the questionnaires were 377 participants, with a response rate of 97%. Among the 377 participants, the majority, 261 (69.2%), were rural residents. The mean age of the participants was 27.5 years (SD =  ± 6.1 years). Regarding educational status, 167 (44.3%) could read and write, 61 (16.2%) attended high school, and 34 (9%) attended college and above (Table 1).

**Table 1:** Socio-demographic characteristics of revisit clients at family planning clinics in Toke Kutaye district, 2021 (*n* = 377)

Variable	Category	Frequency	Perent
Age	< 20	37	9.8
	20–24	81	21.5
	25–29	120	31.8
	30–34	77	20.4
	35 and above	62	16.4
Religion	Protestant	250	66.3
	Orthodox	106	28.2
	Muslim	13	3.4
	Other	8	2.1
Ethnicity	Oromo	334	88.6
	Amhara	36	9.5
	Gurage	7	1.9
Marital status	Single	41	10.8
	Married	291	77.2
	Divorced	30	8.0
	Widowed	15	4.0
Educational status of women	Cannot read and write	60	15.9
	Read and write	167	44.3
	Elementary school	55	14.6
	High school	61	16.2
	College and above	34	9.0
Educational status of husband	Cannot read and write	22	5.8
	Read and write	64	16.9
	Elementary school	76	20.1
	High school	76	20.1
	College and above	99	26.2
Occupation	Housewife	166	44.0
	Government employee	49	13.0
	Private business	63	16.7
	Farmer	82	21.8
	Others	17	4.5
Monthly income	< 2000	236	62.6
	> 2000	141	37.4
Place of current residency	Urban	116	30.8
	Rural	261	69.2

### Reproductive history of the participants

The majority, 336 (89.2%), of the respondents had ever given birth. Among those who had ever given birth, 247 (65.5%) had at least one child, 89 (23.6%) had 2 to 4 children, and 41 (10.9%) had 4 or more children. Concerning their future fertility control, 226 (59.9%) of them want to space, and 54 (14.3%) want to limit. At the time of the interview, the majority (303, 80.4%) of the respondents would like to have an additional child (Table 2).

**Table 2:** Reproductive characteristics of revisiting clients at family planning clinics in Toke Kutaye district, 2021 (*n* = 377)

Variable	Category	Frequency	Percent
Age at first marriage	< 18	136	35.9
	> 18	201	53.3
Ever given birth	Yes	336	89.2
	No	41	10.8
Age at first birth	< 20	172	45.7
	> 20	164	43.5
Number of alive children	< 2	247	65.5
	2–4	89	23.6
	> 4	41	10.9
Plan for having children in the future	Yes	303	80.4
	No	74	19.6
Number of children planned in the future	Never want more children	74	19.6
	1–2	113	30.0
	3–4	156	41.4
	5 +	34	9.0
Future fertility control	To space birth	226	59.9
	Depend on husband	47	12.5
	Depend on God	28	7.4
	To stop birth	54	14.3

### Family planning-related characteristics

The participants mentioned health care providers (43.2%) as their main source of information, and 31.4% of them heard from health extension workers. More than three-fourths (75.6%) of the participants choose by themselves the type of family planning they have been using. In addition, 92.5% of their husbands know about their use of long-acting family planning (Table 3).

**Table 3:** Family planning-related characteristics of revisited clients at family planning clinics in Toke Kutaye district, 2021 (*n* = 377)

Variable	Category	Frequency	Percent
Where do you get information about family planning?	Neighbor	29	7.7
	Husband	56	14.9
	Health care provider	163	43.2
	Health extension worker	120	31.8
	Other	9	2.4
Is your Husband aware your use of family planning?	Yes	266	91.4
	No	25	8.5
Who chooses long-acting family planning method for you?	My self	285	75.6
	Husband	70	18.6
	Neighbor	14	3.7
	Health care provider	8	2.1
Do religious leaders in your area support family planning?	Yes	369	97.9
	No	8	2.1

### Long-acting family planning switching

**Fig. 1:**
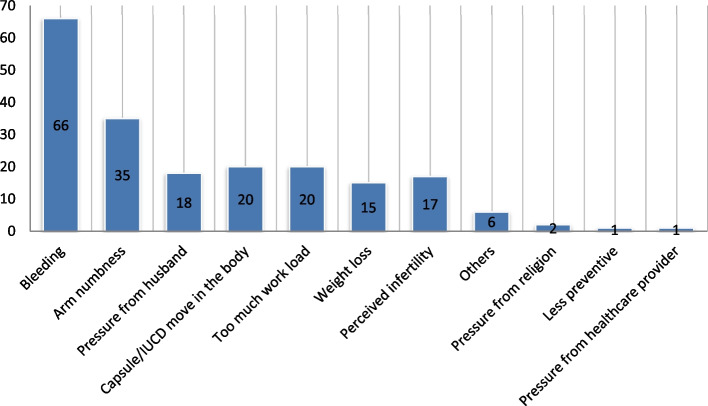
Reasons for long-acting family planning switching among revisit women in Toke Kutaye district, 2021 (*n* = 377)

The study find that 201(53.3%) of the participants switched to different methods. Switching from implants and IUCD to other short-acting methods was 39.8% and 13.5%, respectively. The main reasons for method switching from long-acting method to other methods were bleeding, numbness in the arm, too much work load, Capsule/IUCD move in the body (Fig. 1).

### Phase II: Qualitative findings

A study finding of in-depth interviews from family planning providers stated that LAFP switching is common in their health center despite providing adequate information. Most women returned to the health facility after one month of IUCD insertion due to method’s side effects, lack of adequate information, religion and myths and misconceptions.

### Subordinate themes: Reason for LAFP switching

The findings revealed that fear of side effects, lack of adequate information, religion, and misconceptions caused the users to switch from the long-acting contraceptive method to another.

#### Theme 1: Fear of side effects

A provider at the family planning clinics further described the side effects of LAFP results as switching. Some LAFP users had difficulty maintaining the methods for longer durations. Some users perceived the IUCD has undesirable effects such as fatigue, headache and weight gain. A provider described how method side effects were a barrier to retaining the LAFP:

I remember, the clients told me that ‘’we are a daily labour and we have to work hard. However, this birth control (IUD) deters us from moving and working freely.’’ Different women in the community talk something frustrating about retaining the IUCD. Some of them said that IUCD causes uterine cancer; it is bad when it stays in our uterus for a long period of time. It may affect our sexual desire. In addition, they complained that women in their village who used this method did not give birth. After complaining this, all they shift to short acting. (P05).

A provider shared her experience regarding users’ complaints of implants changes in their menstrual cycle.

“After insertion of Implanon, my period became twice and more in a month and caused me fatigue. Mainly, blurring of my vision became a common problem; hence, it appeared very difficult to continue with it. Therefore, I decided to take Implanon out and shift to pills. (PO3) Nurse.

#### Theme 2: Lack of adequate information about LAFP

A lack of sufficient information on long-acting reversible family planning methods was described by some key informants as another reason for early LAFP method switching. A LAFP provider quoted his experience as follows:

‘‘Almost the majority of our clients do not have adequate information regarding the advantages of long-acting family planning, and they repeatedly complain about the side effects even when they request available types of family planning rather than knowing the detailed advantages and disadvantages of each type. This type of complaint was often heard among educated clients as well. (P04).

On the other hand, FP providers overlooked the benefit of delivering health education on method’s side effects and potential complications. Another provider explained the reason for early LAFP method switching:

Some IUCD providers don’t tell the clients everything about the method. They only tell them the benefits without informing them about its adverse effects and complications. Therefore, when the clients have any adverse effects, they will lose trust in the method as well as in the providers, and they will eventually want to remove it. They won’t remove if they were informed adequately. (P01).

#### Theme 3: Myths and misconceptions

With regard to IUCD switching, some study participants quoted their clients’ reasons, including myths and misconceptions. Some perceived IUCD might affect their fertility in the long term, while others perceived that IUCD might cause stirring in the uterus. Nevertheless, the fairy tale was perceived as a marker of their switch to another contraceptive method. Some IUCD users assumed that the myths of a device may have been moved into the body and switched them to an alternative contraceptive method. A provider explained the reason for early LAFP method switching:

I heard the IUCD can walk to the uterus and heart. I also heard a woman had a baby and the baby was carrying IUCD in his hand. If you get IUCD, you won’t have children again. They have been saying all these things. Additionally, the implant moves to the brain. So I told them, it is not true. (P06).

The study participants reflected that most family planning providers faced challenges due to the women’s poor perception of LAFP. The client's misconception of the IUCD impedes healthcare providers' ability to provide services effectively. Another provider shared their experience regarding LAFP method switching as follows:

They think once is been there for a lone time, most client fear that it may cause infertility. Therefore, they want to remove it and see if they are still fertile and so they will remove, they will get pregnant and then they will come for help. (P02).

Client myths and misconceptions affect IUD provisions. Thus, providers shared their experience regarding LAFP method switching as follows:

The majority of our clients complain of myths from society; for instance, IUCD may cause infertility and damage the uterus by affecting the surrounding tissues of women. (P08)

#### Theme 4: Religion

Religious ambiguity led the clients to be uncertain about the birth control method. Some women perceive using the family planning method as a sin that would result in infertility and regard it as punishment. A provider highlighted how religious faith hinders users from maintaining the LAFP:

Women’s expressed their thoughts on these issues openly. Some challenge you, and tell you that in the Holly Bible, God said ‘you should have many children. They complain that LAFP causes infertility and that it may make God punish us. (P07).

### Factors associated with switching to long-acting family planning

Binary logistic regression was used to assess the association between the dependent and independent variables. Factors that showed a *p* value of < 0.25 were included in the multivariable logistic regression model. The results of the bivariate analysis showed that age of the respondent, educational status of respondents, place of residency, future fertility control, respondents husbands aware of their family planning usage, information received on family planning, fear of using long-acting causes infertility, plan for having children in the future, and age at first marriage were associated with long-acting family planning switching.

The multivariable logistic regression analysis showed that the educational status of women, future fertility control, and fear of infertility were associated with long-acting family planning switching. Switching from LAFP to another method was 10.38 times higher among women who could read and write than among women who attended college and above (AOR, 10.38, 95% CI: 3.481, 30.95). Respondents who wanted to space their birth were 5 times more likely to switch than those who wanted to stop birth (AOR, 5.52, CI, 1.310, 23.33). Respondents who perceived that long-acting family planning causes infertility were 11 times more likely to switch than their counterparts (AOR, 11.16, 95% CI: 5.55, 22.45) (Table 4).

**Table 4:** Multivariable logistic regression analysis of revisited clients at family planning clinics in Toke Kutaye district, 2021 (*n* = 377)

Variable	Switched method		Crude OR (95%CI)	Adjusted OR (95% CI)	*P* value
**Age**	**Yes**	**No**			
15–19	20(54.1)	17(45. 9)	1.525(.673, 3.458)	0.69(.139, 3.450)	0.65
20–24	43(53.1)	389(46.9)	1.467(.754, 2.853)	2.28(.724, 7.209)	0.15
25–29	64(53.3)	56(46.7)	1.481(.799, 2.746)	0.83(.314, 2.201)	1.98
30–34	47(61.0)	30(39.0)	2.031(1.029, 4.007)	1.49(.504, 4.440)	0.14
35 +	27(43.5)	35(56.5)	1	1	
**Educational status**					
Unable to read and write	130(50.0)	30(50.0)	1.429(0.611, 3.342)	1.73(.499, 6.02)	0.39
Read and write	134(80.2)	33(19.8)	5.801(2.654, 12.680)	10.38(3.481, 30.96)*	0.001
Elementary school	10(18.2)	45(81.8)	0.317(.121, .835)	0.94(.248, 3.58)	.093
High school	13(21.3)	48(78.7)	0.387(.155, .969)	0.87(.230, 3.31)	0.84
College and above	1441.2)	20(58.8)	1	1	
**Residence**					
Urban	47(40.5)	69(59.5)	1		
Rural	154(59.0)	107(41.0)	2.113(1.354, 3.298)	0.76(.35,1.65)	0.491
**Future fertility control**					
To space birth	147(92)	85(37.8)	3.529(1.383, 9.006)	5.52(1.31, 23.33)*	0.020
Dependon Husband	11(40.7)	16(59.3)	1.473(.452, 4.798)	2.06(.31, 13. 70)	0.341
Depend on God	20(42.6)	27(57.4)	1.587(.546, 4.615)	4.04(.76, 21.28)	0.562
To stop birth	23(41.1)	33(58.9)	1	1	
**Husband aware of wives’ use of family planning**					
Yes	172(54.8)	140(45.2)	2.45(1.022,5.909)	2.13(.62, 7.26)	0.19
No	8(32.0)	16(68.0)	1	1	
**Received information during LAFP insert**					
Yes	120(44.3)	151(55.7)	1	1	
No	81(76.4)	25(23.6)	4.077(2.452,6.77)	0.24(.108, .54)	0.56
**Plan for having children in the future**					
Yes	169(55.8)	134(44.2)	1.655(2.991, 7.64)	0.62(.19, 2.07)	0.42
No	32(43.2)	42(56.8)	1	1	
**Using family planning causes infertility**					
Yes	146(83.0)	30(17.0)	12.91(7.382, 21.310)	11.166(5.55, 22.45)*	0.001
No	55(27.4)	146(72.6)	1	1	
**Number of children planned in the future**					
Never	329(43.2)	42(56.8)	1	1	
1–2	62(54.9)	51(45.1)	1.596(.88, 2.88)	0.90(.32, 2.55)	0.95
3–4	84(53.8)	72(46.2)	1.53(.877, 2.67)	1.07(.38, 3.02)	0.65
5 +	23(67.6)	11(32.4)	2.74(1.16, 6.44)	2.95(.63,13.75)	0.11

^*^significant at *P* value < 0.05

## Discussion

Overseeing the sustainable use of long-acting family planning methods is a critical intervention to ensure the success of a family planning program. Nonetheless, when women engage in contraceptive switching, it is often associated with unintended pregnancies and eventually leads to unsafe abortions. This study showed that 201 women (53.3%) switched from long-acting family planning methods to another contraceptive method despite wanting to prevent unintended pregnancies. Among these, 39.8% switched from an implant and 13.5% switched from an IUCD. This finding is comparable to other studies in sub-Saharan Africa, such as in Zambia, where LAFP switching was 33% [21]. The finding of this study is relatively high compared to reports from other aforementioned studies in the United States of America [22]. This disparity may be due to different study designs, sample sizes, settings, or study periods.

The reason for switching from the long-acting contraceptive method to other short-acting or traditional family planning methods was explained by quantitative and qualitative data. This study demonstrates that women with low educational status were 10 times more likely to switch LAFP methods than women with higher educational status (AOR, 10.38; 95% CI: 3.48, 30.95). Similarly, a qualitative study in Ghana [23] reported that a lack of education caused women to switch or discontinue IUCD. Furthermore, a study conducted in Uganda [24] revealed that women’s understanding and attitude considerably result in long-acting family planning switching. In contrast to this finding, a study in Senegal [25] showed that women with higher educational status are more likely to switch to the long-acting contraceptive method than those who do not attend formal education. This might be due to the accessibility of information and the ability to comprehend long-acting family planning.

The other rationale for long-acting method switching could be due to a lack of comprehensive information regarding the benefits and adverse effects of implants and IUCDs before insertion by health providers. Several studies have established that the main reasons for LAFP switching and discontinuation among women were its side effects. The findings of this study revealed that respondents who perceived that long-acting family planning causes infertility were 88.88% more likely to switch to other short-acting contraceptive methods than their counterparts (AOR, 11.166, 95% CI: 5.55, 22.45). Conversely, these findings are consistent with retrospective study reports from 36 low- and middle-income countries that demonstrate that among women who utilized long-acting contraceptive methods, 40.2% discontinued because of side effects [26].

Misperception regarding IUCDs and implants among users tend to cause them to switch to other short-acting contraceptive methods. Women’s myths of LAFP methods cause infertility, which causes them to change. This finding is congruent with the studies in Pakistan Khatri and the Democratic Republic of Congo and Chad [27], which reported that fear of infertility influenced the sustainable use of long-acting family planning methods.

Long-acting family planning methods significantly facilitate adequate birth spacing. However, this study revealed that women who were worried about their future fertility plans were more likely to switch to short-acting contraceptive methods than those who did not have any fertility concerns (AOR, 5.528, 95% CI: 1.310, 23.331). Consistent with the literature in Nepal [28], also reports that women’s apprehension regarding fertility concerns influences the coherence of long-acting reversible family planning use. This might have been due to a lack of appropriate knowledge regarding the LAFP method among women, who tend to perceive LAFP methods as interfering with their fertility desires.

On the other hand, this finding is inconsistent with studies conducted in Bangladesh [29], Ethiopia [10] and Kenya [30] that argued that contraceptive method switching was independent of the fecundity plan. Unless women who switch to a long-acting family planning method do not promptly continue with other contraceptive choices, they are at higher risk of unintended pregnancies.

## Conclusions

The study finds that the proportion of women switching from long-acting family planning was relatively higher than in other studies. Women who had formal education, planned to space birth and fear of infertility were found to be significantly associated with long-acting family planning switching. The main reasons for LAFP switching were fear of side effects, lack of adequate information specific to LAFP and myths and misconceptions. The study implies that the provision of quality contraceptive counselling may mitigate the concern of IUD and implant switching. The study also emphasizes that healthcare providers should work to enhance the acceptability of IUD users.

## Strengths and limitations of the study

This study employed both quantitative and qualitative research methods that enabled us to gain insight and comprehensive data from the study participants. Limitations include the cross-sectional nature of this investigation and the qualitative study did not appraise the family planning user’s perceptions and experiences of contraceptive method switching. Furthermore, the study was limited to public health facilities, which influences the generalizability to a larger population.

## Recommendations

The study suggests that family planning service providers should give adequate information and counselling about each contraceptive method of risk and benefit. The study implies that providers should give due accentuate to clients who have complaints with the family planning methods they use. Additionally, the health facilities should engage with the wider community through mass and peer campaign strategies to break myths and misconceptions regarding long-acting family planning. To minimize the effect of the aforementioned limitations, future prospective research at a larger sample size is needed.

## Data Availability

The data sets used during the current study are available from the corresponding author upon reasonable request.

## Abbreviations

AOR: Adjusted Odds Ratio
CI: Confidence Interval
COR: Crude Odds Ratio
EDHS: Ethiopian Demographic Health Survey
IUCD: Intra Uterine Contraceptive Device
LAFP: Long Acting Family Planning
LARC: Long Acting Reversible Contraceptive
OR: Odds Ratio
SPSS: Statistical Package for Social Science
